# Side-by-side biochemical comparison of two lytic polysaccharide monooxygenases from the white-rot fungus *Heterobasidion irregulare* on their activity against crystalline cellulose and glucomannan

**DOI:** 10.1371/journal.pone.0203430

**Published:** 2018-09-05

**Authors:** Bing Liu, Sumitha Krishnaswamyreddy, Madhu Nair Muraleedharan, Åke Olson, Anders Broberg, Jerry Ståhlberg, Mats Sandgren

**Affiliations:** 1 Department of Molecular Sciences, Swedish University of Agricultural Sciences, Uppsala, Sweden; 2 Department of Civil, Environmental and Natural Resources Engineering, Luleå University of Technology, Luleå, Sweden; 3 Department of Forest Mycology and Plant Pathology, Swedish University of Agricultural Sciences, Uppsala, Sweden; USDA Forest Service, UNITED STATES

## Abstract

Our comparative studies reveal that the two lytic polysaccharide monooxygenases *Hi*LPMO9B and *Hi*LPMO9I from the white-rot conifer pathogen *Heterobasidion irregulare* display clear difference with respect to their activity against crystalline cellulose and glucomannan. *Hi*LPMO9I produced very little soluble sugar on bacterial microcrystalline cellulose (BMCC). In contrast, *Hi*LPMO9B was much more active against BMCC and even released more soluble sugar than the *H*. *irregulare* cellobiohydrolase I, *Hi*Cel7A. Furthermore, *Hi*LPMO9B was shown to cooperate with and stimulate the activity of *Hi*Cel7A, both when the BMCC was first pretreated with *Hi*LPMO9B, as well as when *Hi*LPMO9B and *Hi*Cel7A were added together. No such stimulation was shown by *Hi*LPMO9I. On the other hand, *Hi*LPMO9I was shown to degrade glucomannan, using a C4-oxidizing mechanism, whereas no oxidative cleavage activity of glucomannan was detected for *Hi*LPMO9B. Structural modeling and comparison with other glucomannan-active LPMOs suggest that conserved sugar-interacting residues on the L2, L3 and LC loops may be essential for glucomannan binding, where 4 out of 7 residues are shared by *Hi*LPMO9I, but only one is found in *Hi*LPMO9B. The difference shown between these two *H*. *irregulare* LPMOs may reflect distinct biological roles of these enzymes within deconstruction of different plant cell wall polysaccharides during fungal colonization of softwood.

## Introduction

Fungal colonization of wood is associated with decomposition of plant biomass to sustain the growing mycelia. The major constituent of woody biomass is secondary cell walls, of which the three major components (cellulose, hemicellulose and lignin) form a complex and rigid structure, in which cellulose is densely packed into microfibrils, which in turn are covered by hemicellulose and lignin [1,2]. The inherent recalcitrance of woody biomass against microbial degradation requires wood-colonizing fungi to produce a diverse array of hydrolytic and oxidative enzymes that act in synergy to degrade individual components of the plant cell walls [3]. The enzymatic degradation of plant cell walls not only removes physical barriers to enable fungal colonization of the host plant but also releases free sugars that can be used as a source of metabolic carbon and energy by the fungus [4]. White-rot fungi is the only subclass among wood-decaying fungi that are able to efficiently degrade both the carbohydrate and lignin fractions of woody biomass [5].

The basidiomycete white-rot fungus *Heterobasidion annosum* (Fr.) Bref. *sensu lato* (s.l) is a serious plant pathogenic species complex that causes severe root rot on conifer trees within the boreal and temperate regions [6–8]. The economic losses due to *H*. *annosum* s.l. infection for European forest owners due to tree growth reduction and devaluation of timber at harvest reaches at least 790 million euros per year [9]. The *H*. *annosum* s.l. complex is a group of five phylogenetically distinct species with partly overlapping geographic distribution and host preferences [9–11]. The species *H*. *irregulare* is native within North America where it colonizes and decays mostly softwood trees, such as pine and juniper species [10,12]. *H*. *irregulare* infects fresh wood via basidiospores that land on the exposed stump surfaces or wounds on stem, and spread from one tree to another via root-to-root contacts [13]. The mycelium of *H*. *irregulare* spreads mainly through the heartwood and sapwood of softwood plants, in which the major carbohydrate components of the secondary plant cell walls are cellulose and glucomannan [14].

The depolymerization of wood polysaccharides by fungal systems is known to involve a complex and well-orchestrated enzymatic cascade [15]. The *H*. *irregulare* genome encodes 305 putative carbohydrate-active enzymes [16]. Global gene expression analysis of *H*. *irregulare* growing on woody biomaterials revealed that 27 of these 305 genes were up-regulated more than 10-fold compared to when the fungus was cultured in a glucose-containing liquid medium [16]. About half of the up-regulated genes (14 out of 27) were categorized to encode either putative glycoside hydrolases (GHs) or lytic polysaccharide monooxygenases (LPMOs), both of which have been identified as important enzymes responsible for microbial degradation of plant cell wall polysaccharides [16]. In another study, *H*. *irregulare* was grown on woody biomass, and the most abundant protein by far in the culture filtrate was identified as cellobiohydrolase *Hi*Cel7A [17]. It is the only family 7 glycoside hydrolase in the genome of *H*. *irregulare* and it consists of a single catalytic domain without attached linker region and carbohydrate-binding module (CBM). The structure of *Hi*Cel7A has been determined and the structure was found to be similar to that of other know GH7 CBHs [17]. It is the only cellulase from *H*. *irregulare* that has been biochemically characterized so far.

LPMOs are copper-dependent enzymes that employ an oxidative mechanism to cleave linkages in polysaccharides [18–20], and they have been found to oxidize at either the C1 or the C4 position of pyranose rings of polysaccharides [18,21–23]. The catalytic mechanism of LPMOs require several co-factors including external reducing agent, and reactive oxygen species such as molecular oxygen or hydrogen peroxide [19,21,24,25]. The current categorization divides LPMOs into six auxiliary activity families (AA9, AA10, AA11, AA13, AA14 and AA15) [26–29]. The LPMOs in AA9 family are only found in fungi, including ascomycetes and basidiomycetes [26]. Over the last 10 years, extensive research efforts have been made to understand the biological contribution of LPMOs in plant biomass degradation [30,31]. AA9 LPMOs have been shown to possess different substrate preferences in terms of activities against cellulosic and hemicellulosic substrates, indicating that LPMOs could be recruited for degradation of different polysaccharide components in plant cell walls [18,26,32–37]. They have also been shown to boost the hydrolytic activity of cellulases in terms of saccharification of lignocellulosic substrates [27,38–44], and several AA9 LPMOs show effects of separating fibrils from cellulose fibrillar structures, pointing at important roles in cellulose decrystallization [45–47]. Furthermore, studies with atom force microscopy have revealed that modification of highly resistant microfibril bundles by oxidative cleavage using a C1-oxidizing LPMO could enhance the digestion of crystalline regions by a processive cellobiohydrolase I, providing insights into synergistic cooperation between LPMO and cellulase enzymes [45].

The genome of *H*. *irregulare* has been sequenced and annotated, and was found to encode ten putative AA9 LPMOs [16]. More recently, two putative AA14 LPMOs were also discovered in its genome (GenBank ID: XP_009545121.1 and XP_009545122.1) [27]. All of the ten *H*. *irregulare* AA9 LPMOs are predicted to have an N-terminal catalytic domain, three of them have an additional C-terminal family 1 cellulose binding module (CBM 1), and one has a C-terminal extension without known function [16]. Among the *H*. *irregulare* AA9 LPMOs, *Hi*LPMO9B showed the highest transcription level when the fungus was grown on pine heartwood, and *Hi*LPMO9I when the fungus was grown on the material from the cambial zone of necrotic bark, which suggest that these two LPMOs may play important roles during colonization processes of this fungus on pine trees [16]. Therefore, it is of interest to further understand how these two LPMOs are involved in the depolymerization of plant-cell-wall polysaccharides. We have previously reported the recombinant expression and biochemical characterization of both *Hi*LPMO9B and *Hi*LPMO9I, and the three-dimensional structure and molecular dynamics studies of *Hi*LPMO9B [48]. In brief, *Hi*LPMO9B does not contain a cellulose binding module (CBM) and cleaves cellulose by oxidation at the C1 position of cellulose [48,49], whereas *Hi*LPMO9I is a bi-modular protein with linker-CBM attached to the catalytic domain, and this enzyme oxidizes cellulose at the C4 position, and *Hi*LPMO9I is also found to be active on glucomannan [49].

In this study we present a side-by-side comparison of *Hi*LPMO9B and *Hi*LPMO9I in terms of activity on bacterial microcrystalline cellulose (BMCC) and cooperation with the *H*. *irregulare* glycoside hydrolase family 7 cellobiohydrolase *Hi*Cel7A, as well as their glucomannan activity and detailed analysis of glucomannan products generated by *Hi*LPMO9I and structural factors of *Hi*LPMO9I important for glucomannan binding.

## Materials and methods

### Substrates

Bacterial microcrystalline cellulose (BMCC) was prepared by acid hydrolysis of bacterial cellulose from *Acetobacter xylinum*, extracted from commercially available Nata de Coco as previously described [50]. 20 ml of 2.5 mg/ml BMCC was heated at 95°C in 1 M hydrochloric acid for 1 h. After cooling down to room temperature, the BMCC samples were centrifuged at 4,000 ×g for 5 min and suspended with 20 ml deionized water for 5 cycles. The BMCC was finally resuspended in 20 ml of 50 mM sodium acetate buffer, pH 5.0, and the dry mass content of BMCC was measured using Precisa XM60 moisture analyzer (Precisa Gravimetrics AG, Dietikon, Switzerland). 10 mg/ml konjac glucomannan low viscosity (Megazyme, Wicklow, Ireland) was dissolved in 50 mM sodium acetate buffer, pH 5.0.

### Enzyme preparation

The *H*. *irregulare* LPMOs, *Hi*LPMO9B (Uniprot ID: W4KMP1) and *Hi*LPMO9I (Uniprot ID: W4K8M0), were heterologously expressed in *Pichia pastoris* and purified as described previously [48,49]. The wild-type *H*. *irregulare* Cel7A (*Hi*Cel7A, Uniprot ID: W4KCY5) was produced by growing *H*. *irregulare* in liquid cultivation medium containing microcrystalline cellulose substrate Avicel PH-101 as carbon source (Sigma-Aldrich, Steinheim, Germany), and *Hi*Cel7A was purified from the culture supernatant as described previously [17]. A GH family 7 endoglucanase (*Mt*EG7) from *Myceliophthora thermophila* (Uniprot ID: G2QCS4) was heterologously expressed in *P*. *pastoris*, and the protein was purified as described previously [51]. *Neurospora crassa* LPMO9C (*Nc*LPMO9C, Uniprot: Q7SHI8) was produced as described by Borisova, *et al* [52].

### Microcrystalline cellulose activity assay

To investigate the cooperation between the two LPMOs and the *H*. *irregulare* cellobiohydrolase on degradation of crystalline cellulose, reactions were carried out by incubating 100 μl of 1 mg/ml BMCC with LPMO-CBH mixtures at different molar ratios (*Hi*Cel7A: *Hi*LPMO9B or *Hi*Cel7A: *Hi*LPMO9I = 100:0, 75:25, 50:50, 25:75, 0:100) at a constant total enzyme concentration of 0.5 μM, in 50 mM sodium acetate buffer, pH 5.0, in the presence of 1 mM pyrogallol (Sigma-aldrich, Steinheim, Germany), with three biological replicates for each reaction. The reaction mixtures were incubated at 20°C in a thermomixer (Eppendorf, Hamburg, Germany) with orbital shaking at 700 rpm for 48 h. The reactions were stopped by heating the sample at 95°C for 3 min. The insoluble residues of BMCC were collected by centrifugation of the sample at 13,000 ×g for 5 min, and the supernatant was vacuum-filtrated through 0.45 μm polyethersulfone membranes on a filter plate (Pall Corporation, Ann Arbor, MI, USA). The soluble sugars in the liquid fraction were analyzed and quantified using high-performance anion-exchange chromatography (HPAEC) (detailed procedures as described further down). For the reaction with CBH-LPMO ratio at 0:100, the supernatant was subjected to an extra digestion with 0.5 μM *Hi*Cel7A at 20°C for 1 h prior to HPAEC analysis.

The insoluble residues of BMCC were recovered from the reactions with CBH-LPMO ratio at 0:100 to investigate the actions of CBH on LPMO-modified crystalline cellulose surface. The BMCC residues were re-suspended to 100 μl in 50 mM sodium acetate buffer, pH 5.0, after 3 cycles of centrifugation and dilution with the same buffer. The LPMO-treated BMCC residues were then incubated with 0.5 μM *Hi*Cel7A at 20°C for 6 h, and all the reactions were repeated in triplicate. After heat inactivation and vacuum filtration, the soluble enzyme products were analyzed by HPAEC analysis.

### Glucomannan activity assays

Reduction in the dynamic viscosity of glucomannan by the action of purified *Hi*LPMOs was determined using a Lovis 2000 ME microviscometer (Anton Paar, Graz, Austria). The instrument was equipped with a glass capillary tube of 1.59 mm diameter with a 1.5 mm diameter steel ball inside. The instrument was calibrated with deionized water at 30°C prior to the experiments. Viscosity change was determined as a function of difference in “time of fall” [35] of the steel ball while the capillary was positioned at 60° angle. The capillary with reaction sample was inverted when the ball reached the bottom of the capillary. All reactions were carried out at 30°C for 30 min, in a total volume of 500 μl, with 1 mg/ml glucomannan, and 1 μM LPMO in 50 mM sodium acetate buffer, pH 5.0, in the presence of 1 mM ascorbic acid. *Nc*LPMO9C was used as positive control as it had been shown to decrease the viscosity of glucomannan in a previous study [35]. A sample consisting of substrate and ascorbic acid only was used as negative control. At the end of the 30-min viscosity measurement, 200 μl of the sample was drawn from the capillary and subjected to further depolymerization by incubation with 1 μM *Myceliophthora thermophila* EG7 (*Mt*EG7) at 30°C for 1 h. The depolymerized samples were loaded onto a 1-ml Alltech Carbograph SPE column (Grace Davison Discovery Sciences, Deerfield, IL, USA) and eluted with 30% acetonitrile containing 10 mM ammonium acetate. The eluate was freeze-dried and re-dissolved in deionized water, prior to LC-MS analysis (detailed procedures as described further down) for detection of oxidized products.

### Enzyme product analysis

#### High-performance anion-exchange chromatography (HPAEC)

HPAEC analyses were performed using a CarboPac PA1 2×250 mm analytical column (Dionex Corp., Sunnyvale, CA, USA) in an ICS3000 system equipped with a pulsed amperometric (PAD) detector (Dionex Corp., Sunnyvale, CA, USA) and a 5-μl sample loop. A gradient elution program was applied after a full-loop injection in each analysis. The elution program included the following steps: 0–12 min: 30 mM sodium hydroxide (isocratic), 12–15 min: 30–100 mM sodium hydroxide (linear gradient), 15–40 min: 0–350 mM sodium acetate in 100 mM sodium hydroxide (linear gradient), 40–43 min: 350–1000 mM sodium acetate in 100 mM sodium hydroxide (linear gradient) at a constant flow rate of 0.36 ml/min with a post-column addition of 0.3 M sodium hydroxide at 0.18 ml/min prior to PAD detection.

#### Liquid chromatography–mass spectrometry (LC-MS)

LC-MS analysis was carried out on a Bruker maXis Impact mass spectrometer (ESI-QTOFMS) (Bruker Daltonics, Bremen, Germany) operated in positive mode scanning m/z from 50 to 1500, with calibration using sodium formate clusters. Sample of 10 μl was injected to an analytical hypercarb porous graphitic carbon (PGC) column (Thermofisher; 3 μm particle size; 2.1 mm diameter) connected to a HP1100 LC system (Hewlett–Packard). The separation was performed using a gradient from 0% to 80% acetonitrile in 0.2% formic acid at 0.4 ml/min (0–15 min: 0% to 27.5% acetonitrile (gradient), 15–17 min: 27.5% to 80% acetonitrile (gradient), 17–26 min: 80% acetonitrile (isocratic)). Precursor ions in the ranges m/z 533–541, 695–705, 857–867 were selected and subjected to collision induced (CID) MS/MS for structure determination (m/z = 0–100: 15 eV; 100–500, 25 eV; 500–1000: 30 eV).

### Structural modeling and analysis

A homology model of the *Hi*LPMO9I catalytic domain was built using SWISS-MODEL (http://swissmodel.expasy.org/) using default parameters and the crystal structure (chain A) of *Nc*LPMO9C as template (PDB ID: 4D7U_A; 53% sequence identity). The Ramachandran plots used for validating the *Hi*LPMO9I model was obtained by analysing the model in the PROCHECK analysis server [53] and the quality of the model was assessed by Qualitative Model Energy ANalysis (QMEAN) based on QMEAN score and the Z score obtained [54]. Structure-based alignment of the LPMOs was generated using the program Chimera version 1.11 (http://www.cgl.ucsf.edu/chimera/), using alignment algorithm Needleman-Wunsch and matrix BLOSUM-62 (Gap opening = 12, Gap extension = 1), and the visualization of the protein structures were performed using PyMol molecular graphing software version 1.8.6.0 (DeLano Scientific LLC, San Carlos, USA).

## Results

### The effects of *Hi*LPMO9B and *Hi*LPMO9I on CBH activity on microcrystalline cellulose

**Fig 1:**
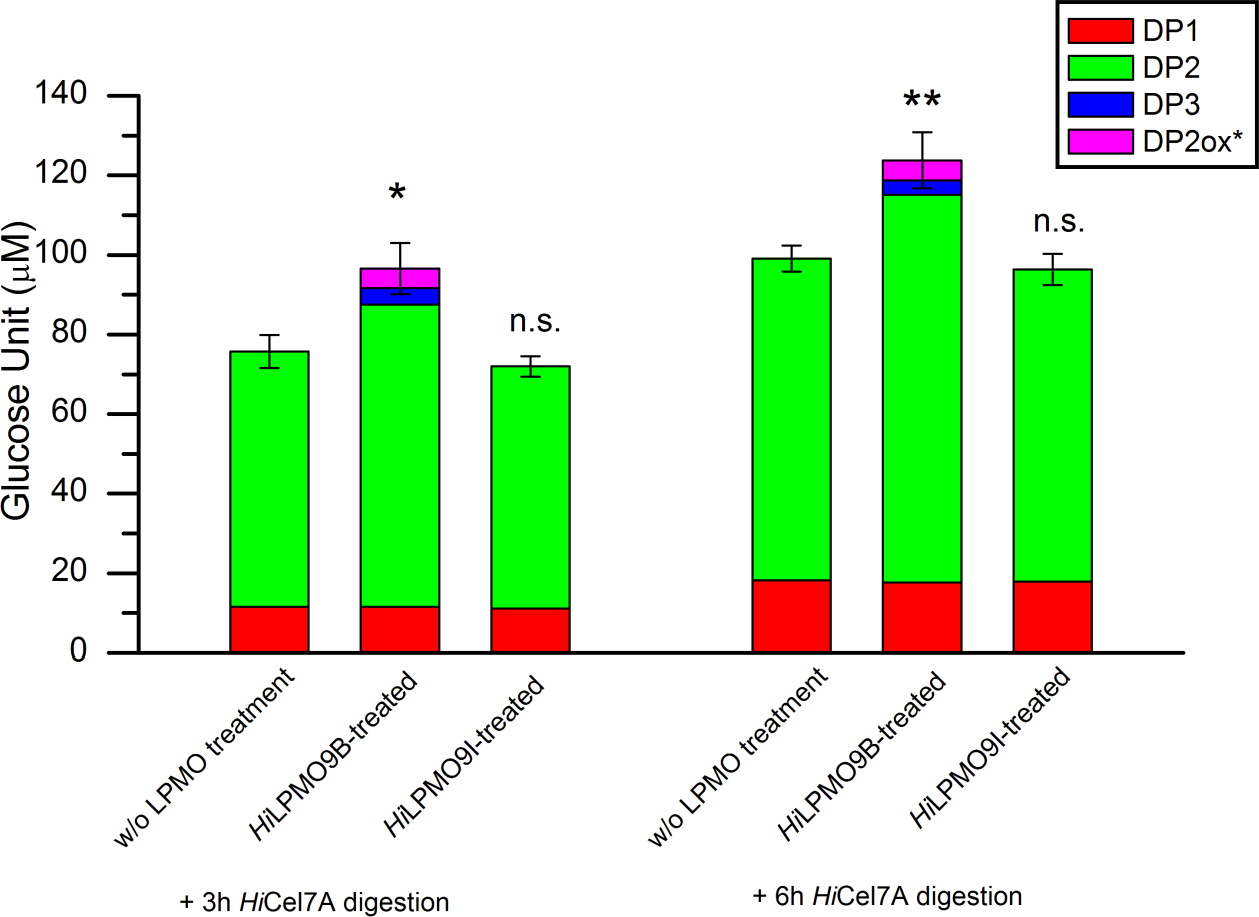
*Hi*Cel7A activity on BMCC substrate residues with or without prior LPMO pretreatment. Soluble sugar released by *Hi*Cel7A from BMCC substrate residues with or without LPMO pretreatment was analyzed by HPAEC. Quantification was based on 3 h and 6 h digestion with 0.5 uM *Hi*Cel7A of the BMCC residue after 48 h pretreatment with 0.5 μM *Hi*LPMO9B or *Hi*LPMO9I in presence of 1 mM pyrogallol (LPMO-treated), or with only pyrogallol without LPMO added as negative control (w/o LPMO treatment). Incubations were done at 20°C, pH 5.0. DPn stands for native cello-oligosaccharides, and DP2_ox_* represents C1-oxidized products (DP1: glucose, DP2: cellobiose, DP3: cellotriose, DP2_ox_*: C1-oxidized cellobiose). Values are means of triplicates, and the error bars show the standard deviations (error bar = ± standard deviation). Two-tailed t-test indicates that total soluble sugar release of *Hi*Cel7A on *Hi*LPMO9B-treated BMCC significantly differ (* p<0.05, ** p<0.01) from the amount on BMCC without LPMO preteatment, but there is no significance difference (n.s. p>0.05) between *Hi*LPMO9I-treated BMCC and BMCC without LPMO pretreatment.

Bacterial microcrystalline cellulose at 1 mg/ml concentration was incubated for 48 h at 20°C, pH 5.0, without and with the addition of 0.5 μM of *Hi*LPMO9B or *Hi*LPMO9I, in the presence of 1mM pyrogallol as reducing agent; thereafter the residual cellulose was washed to remove soluble sugars. The insoluble residue of BMCC was then used to study the release of soluble sugar by 0.5 μM *Hi*Cel7A from the LPMO-modified cellulose sample after 3 h and 6 h of digestion at 20°C, pH 5.0, and thereafter quantified using HPAEC. The pretreatment with *Hi*LPMO9B led to approximately 25% increase in total sugar release (including native and oxidized sugar forms) after 3-h and 6-h digestion with *Hi*Cel7A, whereas no considerable difference was found between *Hi*LPMO9I-treated BMCC compared to BMCC without prior LPMO treatment (Fig 1). There was no change in the level of glucose released between samples with and without prior LPMO treatment. The pretreatment with *Hi*LPMO9B gave 18% more cellobiose than without LPMO after 3 h *Hi*Cel7A digestion, and 20% after 6 h. Also, C1-oxidized cellobiose and cellotriose were detected after incubating *Hi*LPMO9B-treated BMCC with *Hi*Cel7A, but not with the other samples (Fig 1).

**Fig 2:**
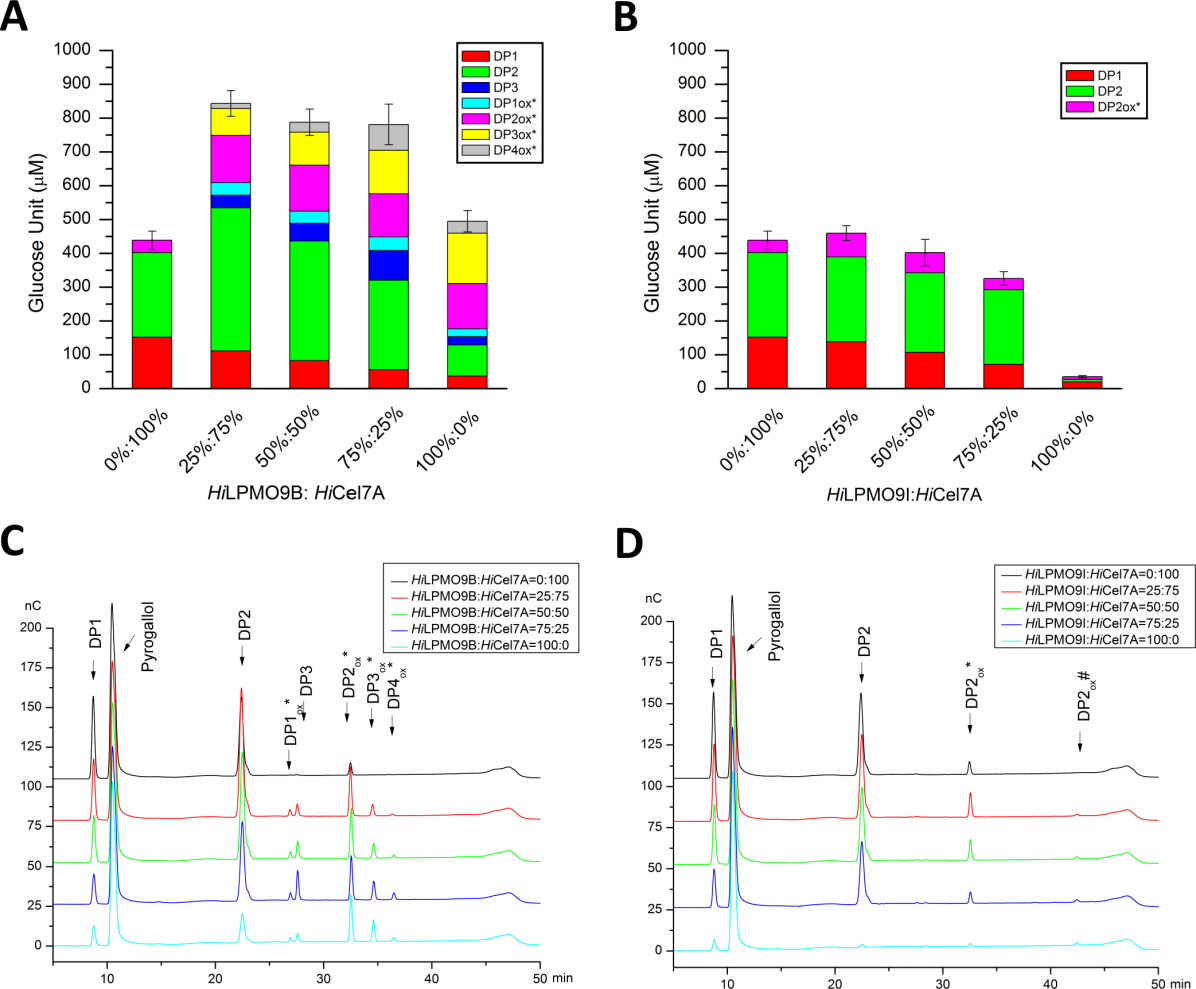
Saccharification levels after treatment of BMCC with LPMO and *Hi*Cel7A at different mixing ratios. Released soluble sugars were analyzed and quantified using HPAEC analysis after 48-h incubation at 20°C, pH 5.0, of 1 mg/ml BMCC with 0.5 μM of *Hi*Cel7A and *Hi*LPMO9B (A) or *Hi*Cel7A and *Hi*LPMO9I (B) at indicated ratios, in the presence of 1 mM pyrogallol as reducing agent. DP1-3 indicate native cello-oligosaccharides and DP1-4_ox_* represent C1-oxidized sugars. The amounts of C4-oxidized sugars were negligible and were not quantified. Values are means of three biological replicates, and error bars correspond to the standard deviation (error bar = ± standard deviation). The quantification is based on HPAEC analysis of soluble sugars released from BMCC by the different combinations of *Hi*Cel7A+ *Hi*LPMO9B (C) or *Hi*Cel7A+ *Hi*LPMO9I (D). DP 1–3: native cello-oligosaccharides; DPn_ox_* represent C1-oxidized products, and DPn_ox_# stands for C4-oxidized products.

Possible LPMO-cellobiohydrolase cooperation on cellulose was investigated by comparing the saccharification of 1 mg/ml BMCC incubated for 48 h at 20°C, pH 5.0, with 0.5 μM mixtures of *Hi*Cel7A+*Hi*LPMO9B, or *Hi*Cel7A+*Hi*LPMO9I, and with 1 mM pyrogallol as reducing agent. Released soluble sugars were analyzed and quantified by HPAEC-PAD (Fig 2). The results reveal that the two LPMOs are strikingly different (Fig 2). *Hi*LPMO9I displayed very little activity, whereas *Hi*LPMO9B actually released more soluble sugar than *Hi*Cel7A when the enzymes were acting alone on the BMCC substrate. The amount of sugar released by *Hi*LPMO9B is about 15 times higher than for *Hi*LPMO9I. Also, there does not seem to be any cooperative effect when replacing *Hi*Cel7A with *Hi*LPMO9I at different ratios; the 0:100, 25:75 and 50:50 replacement experiments are all within standard error (Fig 2B). On the other hand, for *Hi*LPMO9B there is very clear cooperation. The substitution of *Hi*Cel7A with *Hi*LPMO9B significantly boosted the overall saccharification level at all three substitution ratios (Fig 2A). When 25% of *Hi*Cel7A was replaced with *Hi*LPMO9B there was an increase of the total sugar release by 92% compared to *Hi*Cel7A alone, and by 70% compared to using *Hi*LPMO9B alone (Fig 2A). The saccharification levels are similar at 50% and 75% replacement level with *Hi*LPMO9B, giving approximately 80% more soluble sugar than for *Hi*Cel7A alone (Fig 2A). As expected, the amount of oxidized oligosaccharides increased with 25% added *Hi*LPMO9B, but there was also an increase in the release of cellobiose. The proportion of oxidized oligosaccharides is further increased and the amount of cellobiose decreased as the *Hi*Cel7A is further replaced by *Hi*LPMO9B (Fig 2A).

### The activity of *Hi*LPMOB and *Hi*LPMO9I activity on glucomannan

**Fig 3:**
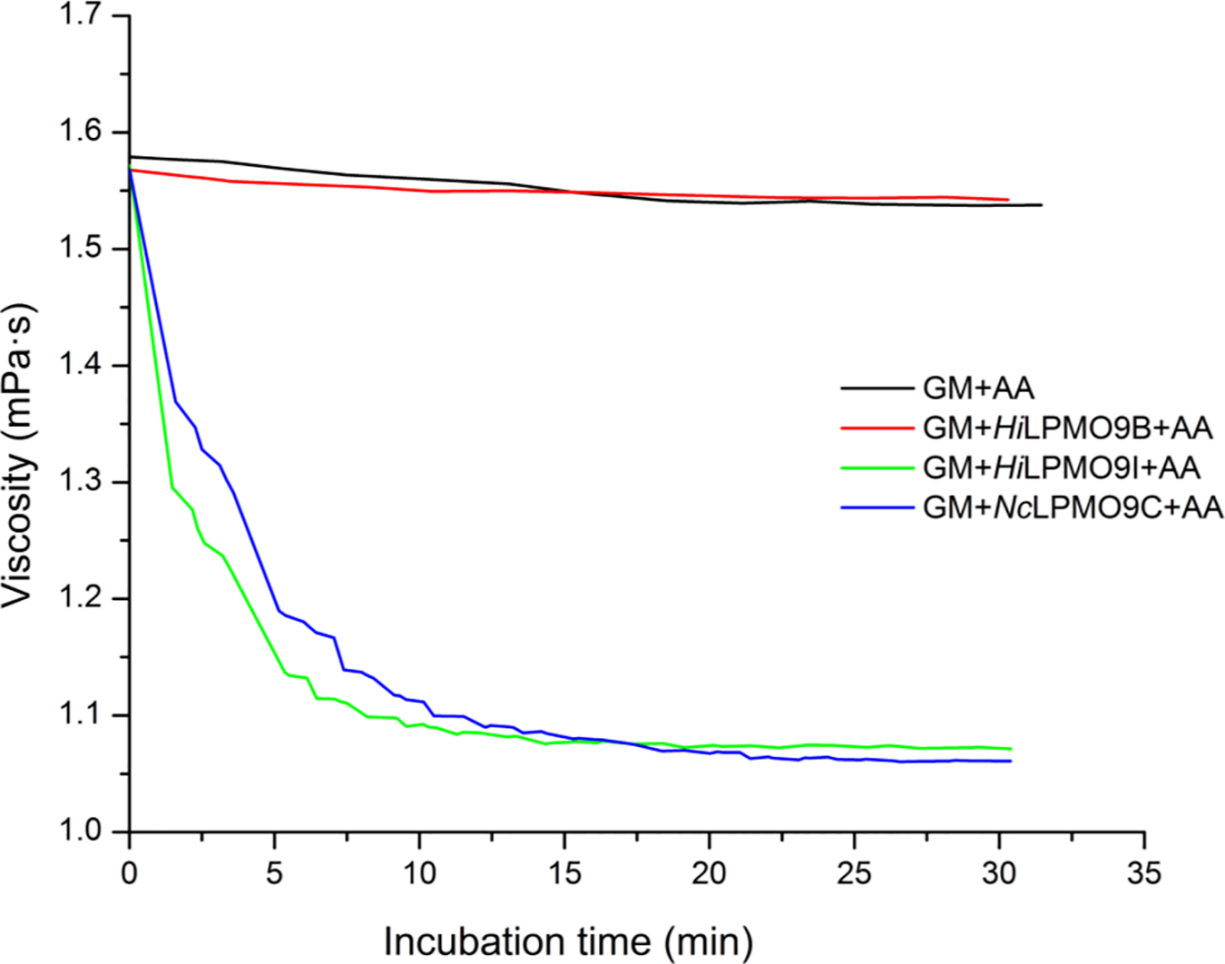
Viscosity changes of glucommanan during the incubation with two *H*. *irregulare* LPMOs using ascorbic acid as reducing agent. The dynamic viscosity was monitored for 30 min at 30°C, pH 5.0, in a falling-ball capillary viscometer with 1 mg/ml glucomannan, 1 uM LPMO and 1 mM ascorbic acid. *Nc*LPMO9C, a LPMO known to oxidatively cleave glucomannan, was used as a positive control, and a negative control containing only glucomannan (GM) and ascorbic acid (AA) was included.

The glucomannan degrading activities of *Hi*LPMO9B and *Hi*LPMO9I were compared in the presence of ascorbic acid as reducing agent using a dynamic viscosity assay (Fig 3). The treatment of glucomannan with *Hi*LPMO9I led to a significant viscosity reduction of glucomannan. The viscosity was reduced by 30% after 15 min incubation with *Hi*LPMO9I, similar to the effect of the positive control *Nc*LPMO9C, which is known to be active on glucomannan [32,35]. Meanwhile, *Hi*LPMO9B did not show any effect on the viscosity of glucomannan and exhibited similar behavior as the negative control without enzyme added.

#### Product analysis after treating glucomannan with LPMOs

**Fig 4:**
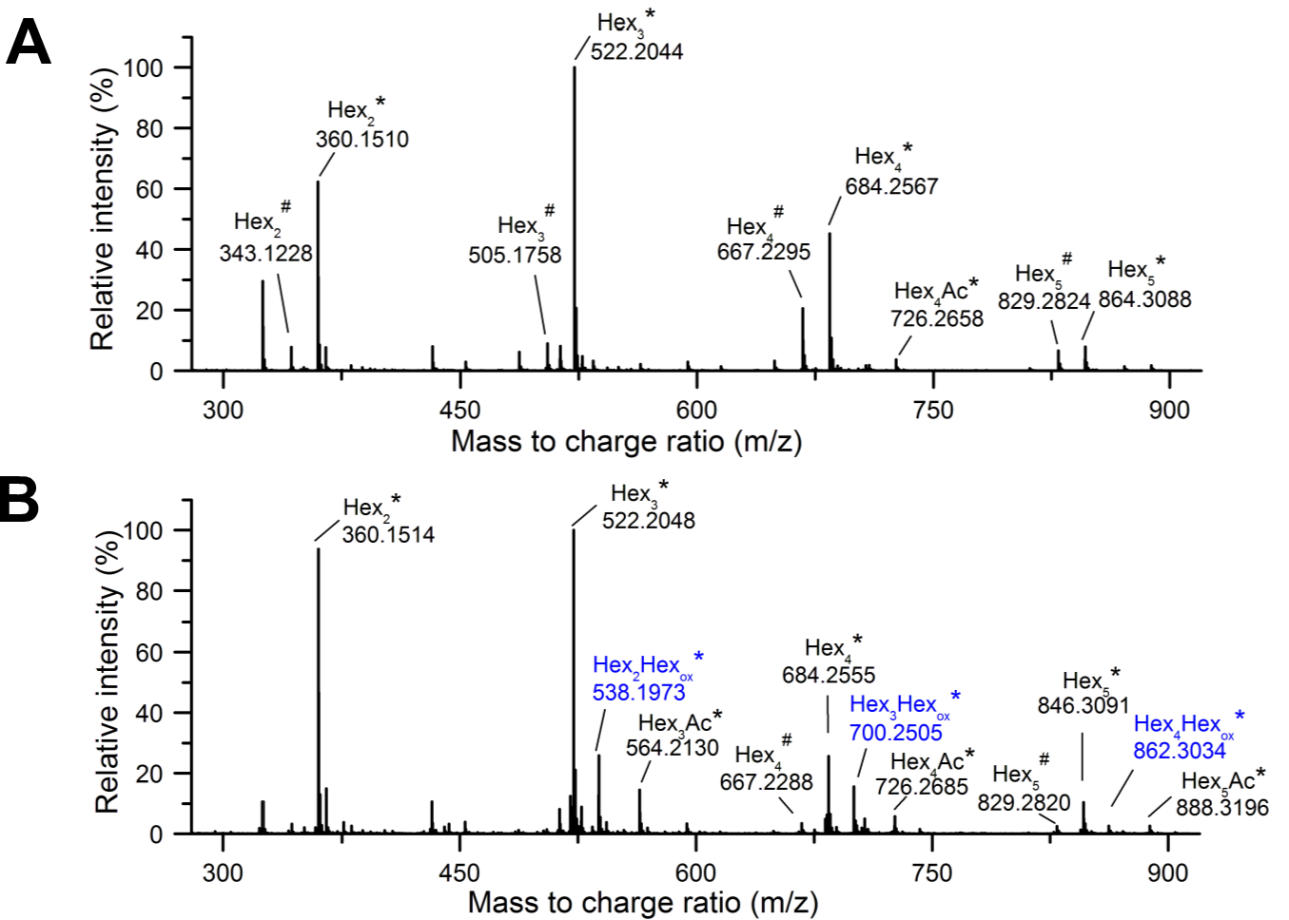
LC-MS analysis of oligosaccharides from LPMO-treated products from glucomannan. Mass spectra are presented as averages over 7.5 to 15 min of the chromatographic analysis of glucomannan treated with *Hi*LPMO9B (A) or *Hi*LPMO9I (B) in presence of ascorbic acid, and subsequent depolymerization with *Mt*EG7. Native (black) and oxidized (blue) glucomannan oligosaccharides were mainly detected as ammonium adducts (*) and to a much less extent as proton adducts (^#^) in the MS analyses.

After the 30 min viscosity measurements, samples of the LPMO-treated glucomannan were withdrawn and depolymerized with an endoglucanase enzyme (*Myceliophthora thermophila Mt*EG7) prior to analysis by LC-MS for detection of oxidized saccharide products. For the *Hi*LPMO9B-treated glucomannan, no oxidized sugars could be detected. Ammonium adducts of native non-acetylated oligosaccharides were detected, with mass-to-charge ratios (m/z) = 360, 522, 684, 846; degree of polymerization (DP 2–5), as well as proton adducts at lower levels (m/z = 343, 505, 667, 829; DP 2–5) (Fig 4A). The *Hi*LPMO9I-treated glucomannan gave a similar product pattern, but also displayed signals for oxidized saccharides at m/z 538, 700, 862, i.e. with m/z +16 Da compared to the corresponding native sugars (m/z = 522, 684, 846) ranging from DP 3 to DP 5 (Fig 4B).

**Fig 5:**
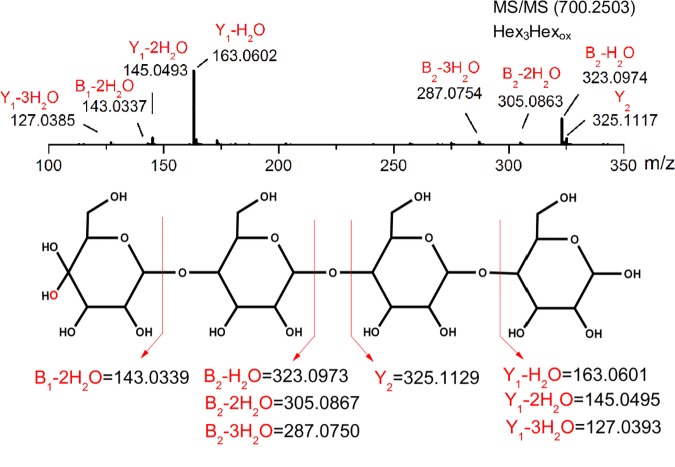
LC-MS/MS analysis of the *Hi*LPMO9I-treated glucomannan sample. MS/MS fragmentation of ion representing oxidized glucomannan tetrasaccharides (upper panel) shows good agreement with the schematic fragmentation pattern of a C4-oxidized gemidiol tetrasaccharides (lower panel).

The oxidized oligosaccharides from the reaction of *Hi*LPMO9I with glucomannan were further studied by MS/MS fragmentation to identify the oxidization position on the pyronose ring. All the three oxidized oligosaccharides, the DP3, DP4 and DP5 saccharides with m/z 538, 700, 862, gave very similar fragmentation patterns (Fig 4). The result of the LC-MS/MS analysis of the oxidized DP4 ion (m/z 700) is displayed (Fig 5). These results point towards the sole existence of C4-oxidized sugars products in the *Mt*EG7-depolymerized sample. The MS/MS fragmentation generated three product ions [m/z = 323.09 (B_2_-H_2_O), 305.05 (B_2_-2H_2_O), 287.07 (B_2_-3H_2_O)] from all the three oxidized GM oligosaccharides, which are in good agreement with B_2_ fragment ions of C4-gemdiols after elimination of water (Fig 5). Additionally, B_1_ fragment ions after elimination of two water (m/z = 143.03) were also observed for DP3 (m/z = 538.1977), DP4 (m/z = 700.2503) (Fig 5). Fragmentation of the ion with m/z 862.3033 (oxidized Hex5) generated a product ion (m/z = 485.15) in line with B_3_-2H_2_O for a C4-gemdiol. Conclusively, C4-oxidized but not C1-oxidized sugars were detected as products from the treatment of glucomannan with *Hi*LPMO9I, and no oxidized sugars at all were detected when the substrate was treated with *Hi*LPMO9B.

#### Structural factors of glucomannan substrate binding

**Fig 6:**
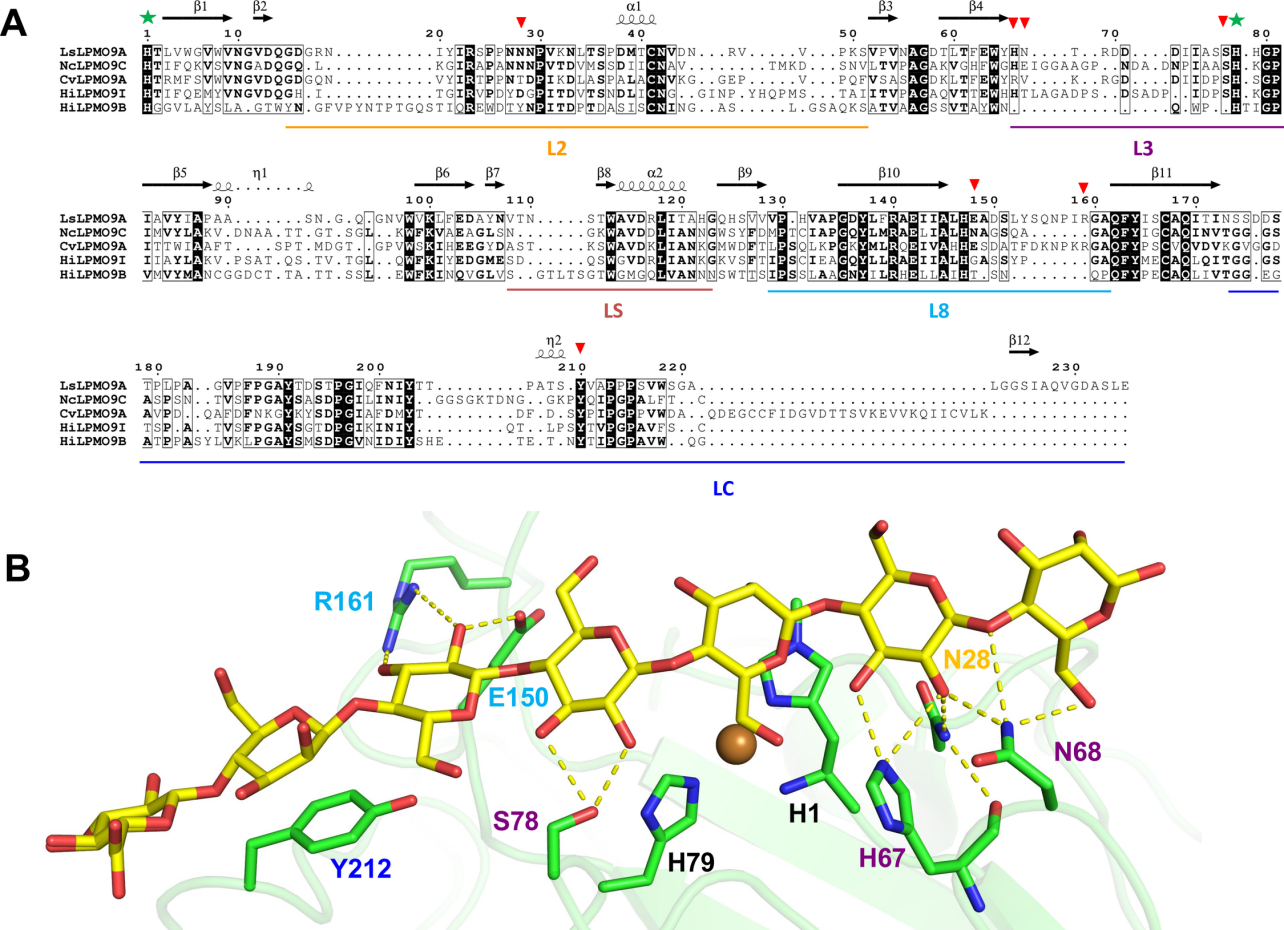
Structural comparison of *Hi*LPMO9B and *Hi*LPMO9I with three other GM-active AA9 LPMOs. (A) Structure-based sequence alignment of *Ls*LPMO9A (PDB code 5NKW; [36]), *Cv*LPMO9A (PDB code 5NLT; [36]), *Nc*LPMO9C (PDB code 4D7U; [55]), *Hi*LPMO9I and *Hi*LPMO9B (PDB code 5NNS; [48]) catalytic domains, and (B) structural detail of GM-oligosaccharide binding to *Ls*LPMO9A (PDB code 5NKW; [36]). The sequence alignment is based on secondary structure information and superposition of the *Hi*LPMO9B structure with the *Hi*LPMO9I homology model, and three other GM-active LPMOs, *Nc*LPMO9C, *Cv*LPMO9A, and *Ls*LPMO9A in complex with glucomannan oligosaccharides. Fully conserved residues are shown in white letters on a black background. Thin-line boxes indicate residues with more than 60% sequence similarity among the aligned sequences. Secondary structure elements of *Ls*LPMO9A are shown at the top of the sequence alignment, where β stands for beta-sheets, α for alpha-helices, and η for 3_10_-helices, respectively. The surface-exposed loop regions are labeled, and underlined in different colors (Loop L2 in gold; L3 in purple; LS in brown; L8 in cyan; LC in blue). The active-site histidine residues of *Ls*LPMO9A are marked with green stars. Red triangles mark residues of *Ls*LPMO9A that were observed to interact with the GM-oligosaccharide in the structure (PDB code 5NKW).

A structure model of the *Hi*LPMO9I catalytic domain, produced using a crystal structure of *Nc*LPMO9C catalytic domain as template (PDB code 4D7U; 53% sequence identity; [55]), was validated with Z score and QMEAN and Ramachandran plot parameters [54]. The QMEAN scoring for model quality estimation indicated a reliable *Hi*LPMO9I model, with Z score of −1.83 and 0.693 as QMEAN score. Ramachandran plot analysis showed 0.6% of amino acid residues in disallowed regions, 97.7% of residues in the allowed region and 1.7% in generously allowed regions [56].The *Hi*LPMO9I structure model was superposed with the crystal structures of *Hi*LPMO9B (PDB code 5NNS; [48]), *Nc*LPMO9C, *Cv*LPMO9A (PDB code 5NLT; [36]) and *Ls*LPMO9A in complex with a GM oligosaccharide (PDB code 5NKW; [36]), to generate a structure-based sequence alignment. The alignment is shown in Fig 6 together with an image of the protein-sugar interactions in the *Ls*LPMO9A/GM-oligosaccharide complex structure. Clearly, *Hi*LPMO9I displays higher similarity with the GM-active *Nc*LPMO9C, *Cv*LPMO9A and *Ls*LPMO9A (53%, 40% and 39% identity, respectively) than does *Hi*LPMO9B (37%, 29% and 28%). *Hi*LPMO9I shares 36% sequence identity with *Hi*LPMO9B. In particular, surface exposed regions of the L2 and L3 loops that interact with GM in the *Ls*LPMO9A structure are more similar in *Hi*LPMO9I than in *Hi*LPMO9B (Fig 6A). One example is the conservative replacement of an asparagine in loop L2 of *Ls*LPMO9A (Asn-28) and *Nc*LMPO9C with an aspartic acid in *Hi*LPMO9I, while there is a hydrophobic tyrosine at the corresponding position in *Hi*LPMO9B. Also, the L3 loop is more similar in length and sequence between these four GM-active LPMOs. The two substrate-interacting residues (His-67 and Ser-78) at either end of the loop are conserved but not Asn-67. Neither of these residues seems to be present in *Hi*LPMO9B structure, which has a much shorter L3 loop. The two GM-interacting residues found on loop L8 of *Ls*LPMO9A, Glu-150 and Arg-161, are not conserved in the GM-active *Nc*LPMO9C and *Hi*LPMO9I (or in *Hi*LPMO9B), but are present in the L8 loop of *Cv*LPMO9A. One interacting residue, Tyr-212 in the LC region, is conserved in all the five aligned sequences, but is also present in most of the known AA9 LPMOs [20], and may thus not necessarily be specific for interaction with glucomannan.

## Discussion

Our results show that the two characterized *H*. *irregulare* LPMOs, *Hi*LPMO9B and *Hi*LPMO9I, are quite different in terms of activity on crystalline cellulose and glucomannan, the major polysaccharides of conifer trees. When BMCC was incubated with the two LPMOs, *Hi*LPMO9I produced very little soluble sugar, in contrast to *Hi*LPMO9B, which even exceeded the *Hi*Cel7A cellobiohydrolase in soluble sugar yield. The difference between these two LPMOs was more pronounced when comparing the saccharification of BMCC by LPMOs and *Hi*Cel7A in conjunction. There was practically no stimulation with *Hi*LPMO9I to *Hi*Cel7A, but clear enhancement was seen when using *Hi*LPMO9B. Mixtures of *Hi*LPMO9B and *Hi*Cel7A, at different ratios but with the same total protein load, gave higher yields of soluble sugar than either of the enzymes on its own. Apparently, *Hi*LPMO9B is complementary in activity and cooperate with *Hi*Cel7A in degradation of cellulose.

The trend was similar when the cellulose sample was treated sequentially, first treated with LPMO and thereafter treated with *Hi*Cel7A. No effect was seen by *Hi*LPMO9I, whereas more cellobiose and also C1-oxidized cellobiose was released by *Hi*Cel7A from the cellulose pretreated with *Hi*LPMO9B, despite the fact that the total amount of remaining cellulose was reduced. Approximately 8% of the cellulose, and presumably the most accessible regions, should already have been degraded and washed away in form of the soluble sugars generated by *Hi*LPMO9B after 48 h incubation. Nevertheless, more soluble sugars could be released by *Hi*Cel7A on the *Hi*LPMO9B-treated BMCC. This indicates that the activity of *Hi*LPMO9B modifies the cellulose substrate to make it more accessible for degradation by *Hi*Cel7A.

These results are in good agreement with the findings in previous studies. It has been shown by AFM that the C1-oxidizing *Neurospora crassa* LPMO9F (*Nc*LPMO9F) caused separation of cellulose fibrils from the crystalline cellulose surface, and the pretreatment of *Nc*LPMO9F could enhance absorption and hydrolytic activity on crystalline cellulose by the cellobiohydrolase *Tr*Cel7A from *Trichoderma reesei* [47]. Similar results were reported in another AFM study wherein the C1/C4-active *Tr*LPMO9A could split larger clusters of crystalline cellulose ribbons into thinner fibrils, and the degradation was accelerated with a combination of *Tr*LPMO9A and *Tr*Cel7A [57]. This is in line with molecular simulations to examine the effect of oxidative cleavage on the structure of crystalline cellulose [58]. As expected, the cleavage due to LPMO actions generated new chain ends that are more solvent accessible. Interestingly, the reducing end was the most solvent exposed of the two new ends at the cleavage site, regardless of which end that was oxidized. LPMO action at either C1 or C4 would thus in both cases primarily increase the exposure of new reducing ends on the cellulose, and potentially the accessibility for reducing end-specific cellulases such as *Tr*Cel7A and *Hi*Cel7A and their productivity.

In contrast to *Tr*Cel7A, the corresponding *Hi*Cel7A does not contain any linker-CBM, but consists of a single GH7 catalytic domain [17]. *Hi*Cel7A is the most secreted enzyme by *H*. *irregulare*, and the only reducing-end specific CBH of *H*. *irregulare*. It is known from previous studies that GH7 CBHs (and also other cellulases) can access much fewer binding sites on cellulose without than with a CBM [59]. Therefore, to increase the number of accessible binding sites on cellulose for *Hi*Cel7A may be even more important in the *H*. *irregulare* system, and suggests that this may be the major role for *Hi*LPMO9B. Indeed, *Hi*Cel7A appears to be even more stimulated than *Tr*Cel7A by the cooperation with an LPMO, although the experiments may not be directly comparable. *Hi*Cel7A is apparently able to attack and cleave off C1-oxidized ends (Figs 1 and 2). We also note that *Hi*Cel7A did generate small amounts of C1-oxidized cellobiose also when acting alone, i.e. in the absence of any LPMO, indicating that C1-oxidized ends were already present in the cellulose substrate, as shown previously [60].

In previous research, nearly all of AA9 LPMOs were characterized using amorphous cellulose (e.g. PASC) as substrate, to determine the regioselectivity on cellulose. The activity of LPMOs on crystalline cellulose has been much less studied, so information is scarce about the relative efficiency of crystalline cellulose degradation between LPMOs with different regioselectivity. In this study, we found that the C1-oxidizing *Hi*LPMO9B is much more efficient in degrading BMCC cellulose than the C4-oxidizing *Hi*LPMO9I.

When glucomannan was used as the substrate the picture was quite different. No activity was detected for *Hi*LPMO9B, whereas clear activity was displayed by *Hi*LPMO9I. So far only five out of 37 characterized LPMOs are known to have activities against glucomannan, but the regioselectivity and substrate specificity of those GM-active LPMOs vary [61]. *Ls*LPMO9A, *Cv*LPMO9A, *Nc*LPMO9C and *Padospora anserina* LPMO9H (*Pa*LPMO9H) displayed broad substrate specificity over a range of hemicellulosic substrates other than GM, such as xyloglucan, mixed-linkage β-glucan, and even xylan in some cases [32,36,37], while *Hi*LPMO9I was only active on GM but not on the other hemicellulosic substrates tested [49]. *Ls*LPMO9A, *Cv*LPMO9A, *Pa*LPMO9H oxidizes both C1 and C4 carbons in glucomannan polysaccharides [32,36,37], while *Nc*LPMO9C only oxidizes at C4 [32,35]. Our MS/MS analysis indicates that similar to *Nc*LPMO9C, *Hi*LPMO9I also oxidizes only at the C4 carbon of the pyranose units in glucomannan. The MS/MS data does not, however, show if the C4 oxidation is on glucose or mannose residues in the glucomannan.

Molecular details of an LPMO with glucomannan was originally shown in the *Ls*LPMO9A-GM complex where the substrate is bound to charged/polar residues located on surface-exposed loop regions (loop L2, L3 and L8) via hydrogen bonding, in addition to hydrophobic interaction with the conserved tyrosine residue on loop LC [36]. Structural comparison of four known GM-active LPMOs (*Nc*LPMO9C, *Ls*LPMO9A, *Cv*LPMO9A and *Hi*LPMO9I) highlights similarities in the L2 and L3 loops in terms of proposed sugar-interacting residues, suggesting that those residues may be essential for glucomannan binding. While 4 out of 7 sugar-interacting residues in *Ls*LPMO9A are conserved in *Hi*LPMO9I, only one (Tyr-212) is maintained in *Hi*LPMO9B. All the corresponding charged or polar sugar-interacting residues on the L2 and L3 loops are missing in *Hi*LPMO9B, which could be a possible reason why no activity on glucomanan was detected for this enzyme. Among the GM-active LPMOs, differences in the suface-exposed loop regions and the distribution of sugar-interacting residues may lead to different positioning of the glucomannan chain relative to the active-site copper ion, which may connect to the varied oxidation preference on glucomannan substrates, but this requires further investigation.

The difference of activity between *Hi*LPMO9B and *Hi*LPMO9I on cellulose and glucomannan indicate possible functional diversification within the LPMO system of *H*. *irregulare*, presumably connected to the biological behavior of this fungus in colonization on softwood. *H*. *irregulare* is known to remove lignin and hemicellulose prior to breaking down cellulose [62], which suggests that deconstruction of glucomannan network may be an initial step that allows hyphal penetration across the plant cell walls. The considerable reduction of glucomannan viscosity shown by *Hi*LPMO9I suggests that this enzyme could be recruited for the disruption of glucomannan network to facilitate the hyphal penetration. *Hi*LPMO9B, on the other hand, is capable of degrading cellulose and increasing substrate accessibility for the *Hi*Cel7A cellobiohydrolase, suggesting that *Hi*LPMO9B could play an important role in crystalline cellulose degradation, through cooperation with other cellulases.

In summary, this study shows that the two *H*. *irregulare* LPMOs, *Hi*LPMO9B and *Hi*LPMO9I, show different performance in terms of degrading glucomannan and microcrystalline cellulose, which suggests that *Hi*LPMO9B and *Hi*LPMO9I could be involved in the different parts of the enzyme machinery used for decomposition of individual polysaccharide components in the softwood cell wall, with different contribution to the colonization process of this fungus on softwood.
